# Outdoor THz fading modeling by means of gaussian and gamma mixture distributions

**DOI:** 10.1038/s41598-023-33598-x

**Published:** 2023-04-19

**Authors:** Evangelos N. Papasotiriou, Alexandros-Apostolos A. Boulogeorgos, Angeliki Alexiou

**Affiliations:** 1 Department of Digital Systems, University of Piraeus Research Center, 18534 Piraeus, Greece; 2 Department of Electrical & Computer Engineering, University of Western Macedonia, 50100 Kozani, Greece

**Keywords:** Electrical and electronic engineering, Computer science

## Abstract

Terahertz (THz) band offers a vast amount of bandwidth and is envisioned to become a key enabler for a number of next generation wireless applications. In this direction, appropriate channel models, encapsulating the large and small-scale fading phenomena, need to be developed for both indoor and outdoor communications environments. The THz large-scale fading characteristics have been extensively investigated for both indoor and outdoor scenarios. The study of indoor THz small-scale fading has recently gained the momentum, while the small-scale fading of outdoor THz wireless channels has not yet been investigated. Motivated by this, this contribution introduces Gaussian mixture (GM) distribution as a suitable small-scale fading model for outdoor THz wireless links. In more detail, multiple outdoor THz wireless measurements recorded at different transceiver separation distance are fed to an expectation-maximization fitting algorithm, which returns the parameters of the GM probability density function. The fitting accuracy of the analytical GMs is evaluated in terms of the Kolmogorov-Smirnov, Kullback-Leibler (KL) and root-mean-square-error (RMSE) tests. The results reveal that as the number of mixtures increases the resulting analytical GMs perform a better fit to the empirical distributions. In addition, the KL and RMSE metrics indicate that the increase of mixtures beyond a particular number result to no significant improvement of the fitting accuracy. Finally, following the same approach as in the case of GM, we examine the suitability of mixture Gamma to capture the small-scale fading characteristics of the outdoor THz channels.

## Introduction

Terahertz (THz) wireless systems have been identified as a key enabler of the next generation networks era, since it can provide the required radio resources for a number of killer-applications, including wireless backhauling, mobile ad-hoc backhauling, as well as massive connectivity of bandwidth-hungry applications, like virtual and holographic reality^1–3^, as well as enabling sensing^4,5^ and cm-level localization capabilities^6^. Novel wireless concepts, such as the internet of everything, connected and autonomous vehicles, and unmanned aerial vehicle, are also expected to benefit from the usage of the THz band^2^. The first step towards designing and optimizing THz wireless systems is the development of indoor and outdoor channel models that can accurately capture the particularities of the propagation medium in this band. In particular, the THz wireless channel model can be seen as the joint contribution of the large and small scale fading^7^. The large scale fading can be expressed in terms of the deterministic pathloss and shadowing, while the fast channel amplitude fluctuations are described in terms of the stochastic small-scale fading^7^.

The large scale fading characteristics have been extensively investigated in both outdoor and indoor environments^8–23^. In more detail, urban outdoor double directional channel measurements in the range of ${141.1{-}148.5\text { GHz}}$ for distances over ${100 \text { m}}$ have been conducted^8^. These measurements by means of the many identified angles of arrival and angles of departure have verified the existence of line-of-sight (LoS) and non-line-of-sight (NLoS) THz multipath components. An outdoor measurement campaign in an urban microcell environment at ${140\text { GHz}}$ recorded omni and directional LoS and NLoS links at a maximum distance of ${117.4\text { m}}$^9^. Based on the aforementioned measurements, omni and directional pathloss exponent models have been implemented, where the shadowing is expressed by means of a lognormal distribution. A ray-based deterministic tool has been employed to model the large scale pathloss of an urban outdoor scenario in the range of ${90{-}200\text { GHz}}$^10^. The pathloss has been modeled by the exponent model, where the shadowing due to vegetation has been modeled by means of a lognormal distribution. Based on ${142\text { GHz}}$ multiple-input-multiple-output (MIMO) urban microcell propagation measurements, the channel spatial statistics of the number of spatial clusters and the cluster power distribution have been identified^11^. A detailed spatial statistical MIMO channel generation procedure has been introduced based on the empirical channel statistics. An extensive set of wireless LoS and NLoS measurements in the range of ${145{-}146\text { GHz}}$ for distances between ${1{-}100\text { m}}$ have been conducted^11^. Building upon the measurements, the deterministic pathloss, shadowing, delay spread, angular spread and multipath component power distribution have been modeled. A vehicle to infrastructure channel has been developed for an urban scenario by means of ray tracing for the operational frequency of ${110\text { GHz}}$^13^. Accordingly, the channel statistics of pathloss, time-of-arrival and direction-of-arrival have been characterized. An initial review on the impact of the weather conditions to the deterministic attenuation of THz wireless links has been conducted^14^. In more detail, the channel impairments caused by the water vapor, dust particles, fog, clouds, and rain have been investigated. Meanwhile, deterministic THz polynomial pathloss models for the ranges of ${100{-}450\text { GHz}}$, ${200{-}450\text { GHz}}$ and ${275{-}400\text { GHz}}$ have been developed^15,18,19^. In these models, the THz channel has been assumed to consist of a single deterministic LoS coefficient, which has been expressed as the sum of the free space and molecular absorption loss. Various LoS and NLoS indoor measurements are performed for wireless links operating at $28\text { GHz}$ and 140 GHz^16,17^. Therein, based on the received signal strength of the multipath components of the links, the millimeter wave (mmWave) and THz channels have been deterministically modeled as the logarithmic scale sum of the exponential pathloss and lognormal shadowing. A single path theoretical THz channel model for THz wireless communications within vegetation has been developed^21,23^. In this model, the wireless channel consists of two coefficients, namely the deterministic pathloss and the lognormal shadowing.

The indoor THz small-scale fading channel modeling has recently gained a momentum^7,16,17,20,24–31^. Specifically, for the case of wireless backhaul THz links, the small-scale fading has been theoretically modeled by means of the ${\alpha {-}\mu }$ distribution^24,26^. Then, the system performance has been quantified under different levels of transceiver hardware impairments, antennas misalignment and fading severity. Furthermore, the suitability of the ${\alpha {-}\mu }$ distribution to describe the small-scale fading channel amplitude of indoor THz wireless channels has been experimentally validated in several studies^7,30,31^. Experimental LoS and NLoS THz wireless measurements have been performed in an anechoic chamber^27^. Based on this model, a stochastic indoor THz channel model has been developed, where the small-scale fading attenuation factor has been expressed in terms of a Rayleigh or Nakagami-m distribution under NLoS and as a Rice or Nakagami-m in LoS propagation conditions, respectively. A two dimensional stochastic geometric channel model has been developed for indoor THz wireless communications^28,29^. Then, a parametric multipath Rice fading model has been derived. A measurement based indoor channel model for the range of ${126{-}156\text { GHz}}$ for both LoS and NLoS conditions has been developed^20^. The exponential pathloss and shadowing have been used to model the large scale fading, whereas the small-scale fading amplitude has been given by a novel distribution. Meanwhile, THz wireless measurements have been conducted within an anechoic chamber in the range of $240{-}300\text { GHz}$^25^. Then, by exploiting the measurements and various fitting accuracy metrics, it has been concluded that the small-scale fading amplitude of the links can be accurately modeled by means of the Gamma and Gaussian mixture models. Also, the mixture Gamma (MG) has been employed in investigating the capacity of a wireless channel and expressions for the optimal and power rate adaptation, the channel inversion with fixed and truncated rate were derived. The expressions were verified by means of Monte-Carlo simulations^32^. Furthermore, the Gamma mixture has been used for analytical performance assessment of composite fading channels in terms of received signal-to-noise-ratio^33^. In continuation of the previously mentioned work, the Gaussian mixture has been employed in the performance analysis of an energy detector. In more detail, analytical expressions for the performance parameters of average detection and area under the receiver probabilities were derived^34^.

The aforementioned contributions underline the importance of not only the large-scale, but also the small-scale fading THz channel modeling. However, to the best of the authors knowledge, results on THz small-scale fading channel modeling in outdoor environments have not been published so far. Motivated by this, in this work, outdoor THz measurements performed in the campus area of Aalto university in Finland are exploited. In more detail, multiple LoS and NLoS links have been measured at different transceiver separation distances. For each link, multiple channel gain measurements were recorded, which have been used to perform fitting analysis of the empirical channel gain distribution amplitude to Gaussian Mixtures (GMs) analytical distributions. The evaluation of the suitability of GMs to describe the small-scale fading channel gain amplitude of outdoor THz wireless links is very useful. An appropriate GM is capable of describing complicated fading scenarios, where multiple peaks can occur in the fading amplitude of the empirical distribution^25,35^. The GM is expressed as the sum of independent Gaussian distributions. Hence, it offers mathematical tractability, which is of great importance in analytical expressions evaluations. By taking this into account it should be noted that, the fluctuating-two-ray (FTR) model has also been employed in THz channel modeling^36,37^. However, the FTR uses an infinite number of components to approximate the empirical distribution. As a consequence simpler distributions like the GM and Gamma mixture are preferred to accommodate the channel modeling and the analytical evaluation needs. Moreover, it should be noted that, in this work the suitability of GMs to model the small-scale fading amplitude of the outdoor THz links is more thoroughly investigated in comparison with MGs distributions. The reason for this is that the analytical expression of the GMs are more tractable in comparison with those of the MGs and have been employed in various performance evaluation works^35,38,39^. Also, the suitability of MG to model the small-scale fading amplitude of short range indoor THz wireless links has been previously investigated^25^. Moreover, the support of a GM is defined in the ${\left( -\infty ,\infty \right) }$, which aids in achieving a good fit to the tails of the empirical distributions.

In this work the measurements of each link are preprocessed to obtain the channel gain of each of the recorded multipath components. Subsequently, in order to increase the number of the different channel realizations in each link, a method based on adding random phases to the path amplitudes will be employed. Then, by making use of the resulting channel realizations of each link, the empirical probability density function (PDF) and cumulative density function (CDF) are fitted to the analytical GMs. Also, MGs distributions are fitted to some indicative links and the fitting performance is compared to that of the GMs. Then, the parameters and weights of each Gaussian and Gamma distribution of a GM and MG expression, respectively, are obtained by fitting it to the empirical channel gain distribution of the investigated link. This is accomplished by means of the expectation maximization (EM) algorithm^25,35,38,40^. The accuracy of the fit of the analytical distributions to the corresponding empirical ones is quantified in terms of the Kolmogorov-Smirnov (KS), Kullback-Leibler (KL) and root-mean-square-error (RMSE) tests^41–43^. However, the evaluation of the fitting accuracy of the analytical GMs and MGs to the empirical ones is performed only in terms of the KL and RMSE tests, because, for all the GMs and MGs of all the investigated links, the KS yields a good fit. As a result, the KS poses as a non strict fitting criterion. According to the KL and RMSE metrics for all the links, it is observed that, as the number of mixtures increases the resulting analytical GMs and MGs perform a better fit to the empirical distributions. On the other hand, as the number of mixtures decreases, the resulting analytical GMs and MGs perform worse in terms of fitting even for single peak empirical distributions. Furthermore, the KL and RMSE metrics indicate that the increase of mixtures above a particular threshold does not improve drastically the fitting accuracy performance of the analytical GMs and MGs to the empirical ones.

In order to further elucidate, the key contribution of this work lies in the approach that is followed to derive the empirical small-scale fading amplitude distribution of the investigated THz links. In more detail, the principle of transfer learning combined with the EM algorithm is employed for the measured data of an outdoor static THz propagation environment^44^. These THz wireless link measurement data contain deterministic pathloss measurements and during each link measurement session there were no moving scatterers. Yet, in a realistic THz wireless signal propagation scenario moving scatterers may influence the channel characteristics. This can be adequately modeled by the methodology initially proposed by Molisch et al.^45^. In this work this methodology is employed to populate the herein used link measurements datasets^44^. Next, after observing the resulting empirical PDF of each measured THz link, we propose the GM distribution as a suitable target distribution. In order to identify the number of Gaussian distributions needed and their corresponding weights and parameters we follow a fitting methodology based on an interactive EM algorithm.

## Results

### Measurement setup and sites

**Figure 1:**
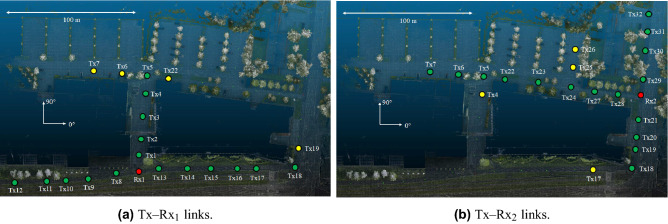
Top-view of the outdoor campus premises.

Figure 1 illustrates the top-view of the outdoor premises of Aalto University in Finland, where the THz measurements are conducted. In more detail, each link is defined by a unique transmitter (Tx) and receiver (Rx) pair. Both the Tx and Rx are equipped with a single antenna. During each measurement session both the Tx and Rx were in fixed positions, while only the Tx-Rx pair of interest was active, i.e., no interference is induced by neighbor links. Figures 1a and b show that individually $\textrm{Rx}_1$ and $\textrm{Rx}_2$ are employed to perform the wireless THz measurements. The Txs marked with green dots denote a LoS link between the Tx and the Rx of interest, whereas the Txs marked with a yellow dot stand for a NLoS transceiver link. However, it should be noted that for the investigated outdoor THz measurements no paths were able to be received in the NLoS transmissions scenarios. The THz transmissions of all the investigated links are performed at the center radio frequency (RF) of ${142\text { GHz}}$ with a total bandwidth of ${4\text { GHz}}$^44^. The transmit power is set equal to $5\text { dBm}$ and the transceivers antennas heights are $1.85\text { m}$. The Rx is equipped with a sectoral horn antenna with a gain of ${19\text { dBi}}$, whereas the Tx is equipped with an omni-directional antenna. Also, during the measurement of each ${\textrm{Tx}{-}\textrm{Rx}}$ link, the Rx antenna is rotated with an angular step of $5^{\textrm{o}}$ and no moving objects are present.

### Fitting of the gaussian & gamma mixtures to the channel gain measurements

**Figure 2:**
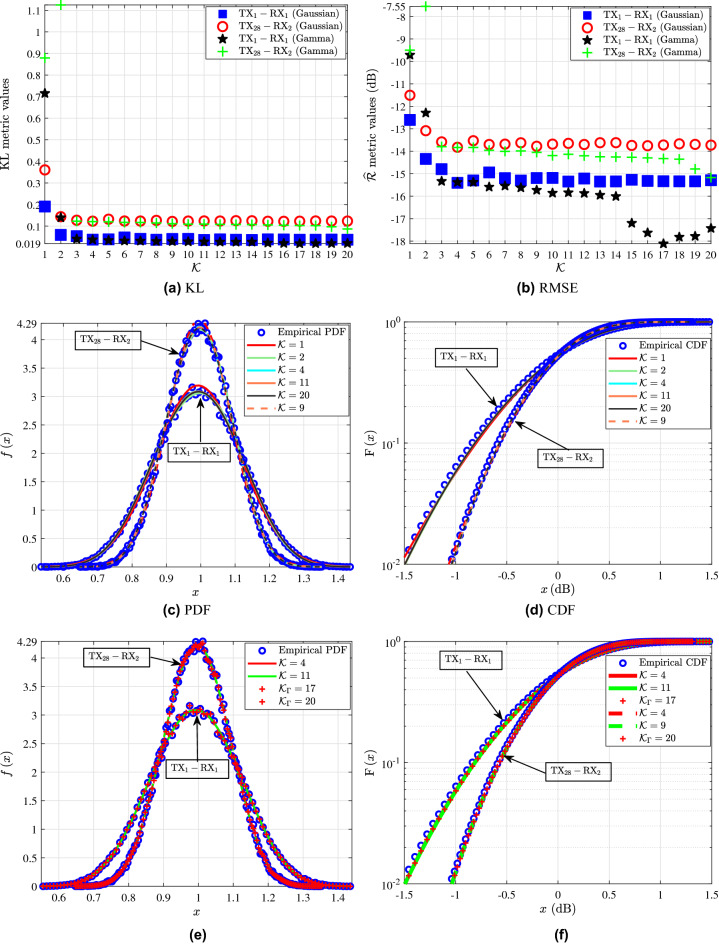
${\textrm{TX}_1{-}\textrm{RX}_1}$ and ${\textrm{TX}_{28}{-}\textrm{RX}_2}$: (**a**) KL and (**b**) $\mathrm {\widehat{R}}$ metrics for different values of *K* for the GMs and MGs. (**c**) Fitting of the PDF and (**d**) CDF analytical GM expressions to the empirical channel gain data. (**e**) Fitting of the PDF and (**d**) CDF of the best fitting analytical GM and MG expressions to the empirical channel gain measurements.

**Figure 3:**
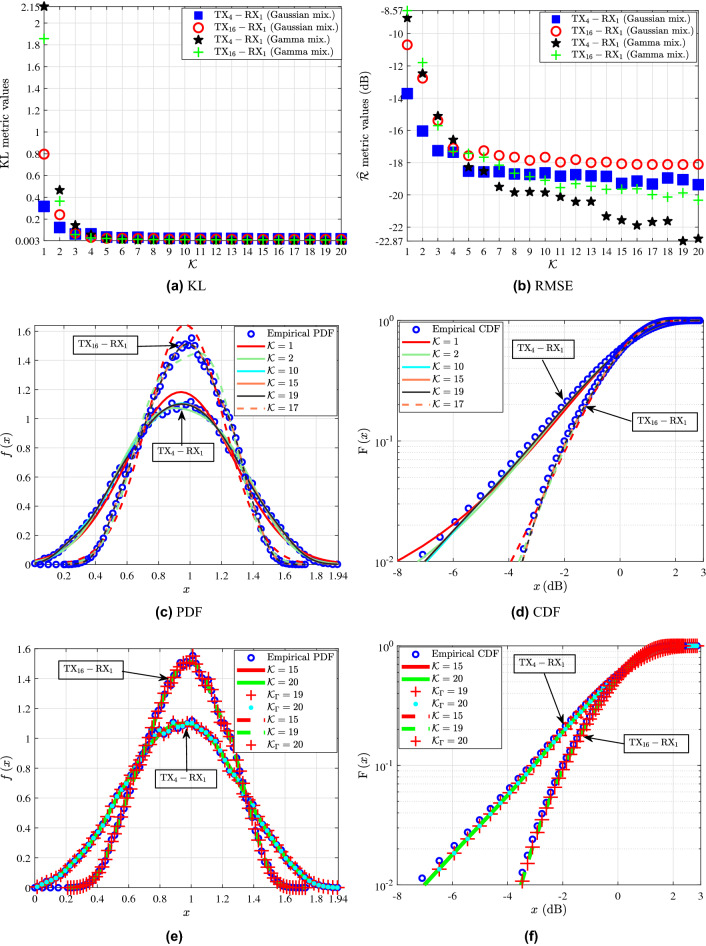
${\textrm{TX}_4{-}\textrm{RX}_1}$ and ${\textrm{TX}_{16}{-}\textrm{RX}_1}$: (**a**) KL and (**b**) $\mathrm {\widehat{R}}$ metrics for different values of *K* for the GMs and MGs. (**c**) Fitting of the PDF and (**d**) CDF analytical GM expressions to the empirical channel gain data. (**e**) Fitting of the PDF and (**d**) CDF of the best fitting analytical GM and MG expressions to the empirical channel gain measurements.

**Figure 4:**
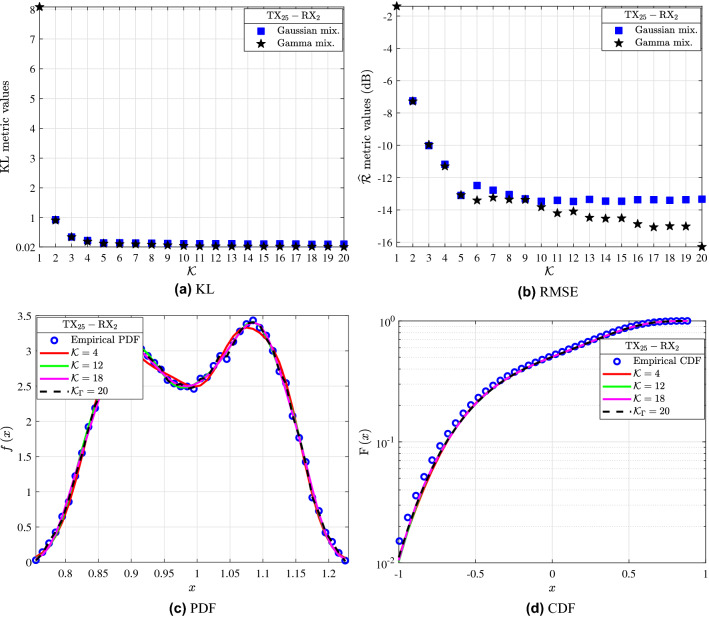
${\textrm{TX}_{25}{-}\textrm{RX}_2}$: (**a**) KL and (**b**) $\mathrm {\widehat{R}}$ metrics for different values of *K* for the GMs and MGs. (**c**) Fitting of the PDF and (**d**) CDF analytical GM and MG expressions to the empirical channel gain data.

**Figure 5:**
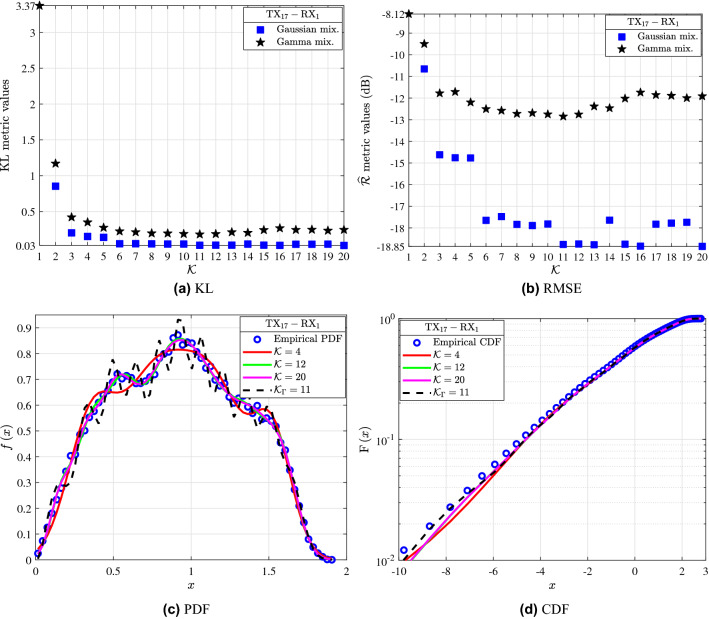
${\textrm{TX}_{17}{-}\textrm{RX}_1}$: (**a**) KL and (**b**) $\mathrm {\widehat{R}}$ metrics for different values of *K*. (**c**) Fitting of the PDF and (**d**) CDF analytical GM and MG expressions to the empirical channel gain data.

In this section, the fading channels are approximated using the GM distribution. Also, some indicative fitting results of modeling the fading channels by means of the MG distribution are presented. In more detail, Figs. 2, 3, 4 and 5 serve as an illustrative example of the fitting achieved by the analytical GMs and MGs expressions, which are obtained as the weighted sum of *K* Gaussian and *K* Gamma distributions respectively, to the empirical channel gain measurements of the investigated links. Table 1 quantifies the fitting achieved by the GMs to the empirical measurements of the links in terms of the KL and RMSE fitting accuracy metrics. The link, $\textrm{d}$, KL, ${\mathrm {\widehat{R}}}$ and *K* columns stand for the ${\textrm{TX}{-}\textrm{RX}}$ link index, the transceiver antennas separation distance, the achieved KL and RMSE metric values and the corresponding *K* of the GM, respectively. The *K* GM components that yield the most accurate fit to the empirical channel gain measurements, are selected by using as a criterion the minimization of the KL metric. Meanwhile, the KS metric for $K \in [1,20]$ for all of the presented links yields a good fit. Hence it is a non strict fitting criterion and cannot be employed to identify the *K* that corresponds to the GM with the best fit to the empirical measurements. Furthermore, the RMSE metric serves as the second best fitting criterion after the KL. Moreover, it should be noted that, the ${K\in [1,20]}$ MGs passed the KS test for all of the examined links. As a result, the KS test cannot be employed to evaluate the fitting accuracy for the MG distributions. Meanwhile, as it can be observed from Figs. 2, 3, 4 and 5 and especially from 2(a)(b), 3(a)(b), 4(a)(b)s and 5(a)(b); the KL and RMSE tests are reliable fitting accuracy tests not only for the GMs but also for the MGs distributions. Note that, for the interested reader the parameters of the GMs and MGs extracted in this work; can be found on the following link: https://github.com/T34gr/Gaussian-and-Gamma-mixture-distribution-parameters.git.

Figure 2 illustrates the statistical characterization of the ${\textrm{TX}_1{-}\textrm{RX}_1}$ and ${\textrm{TX}_{28}{-}\textrm{RX}_2}$ links. In more detail, Fig. 2a shows the KL values of GMs and MGs with different *K* for both ${\textrm{TX}_1{-}\textrm{RX}_1}$ and ${\textrm{TX}_{28}{-}\textrm{RX}_2}$. As expected, for a given link, as *K* increases, the KL value of the GMs generally decreases. After achieving a minimum KL value, as *K* further increases, a short variation towards this value is observed. According to Table 1, for both of the links the maximum KL value is achieved for $K=1$. Meanwhile, for $K=4$ the first local minimum of KL is observed for the GMs of both ${\textrm{TX}_1{-}\textrm{RX}_1}$ and ${\textrm{TX}_{28}{-}\textrm{RX}_2}$, which is equal to 0.037 and 0.123, respectively. For the ${\textrm{TX}_1{-}\textrm{RX}_1}$ link, the global minimum value of KL is achieved for the GM with $K=11$, which can be found in Table 1. On the other hand, for ${\textrm{TX}_{28}{-}\textrm{RX}_2}$ according to Table 1 the global minimum value of KL is achieved for the GM with $K=9$. For the case of MG modeling, from Fig. 2a it is observed that for both ${\textrm{TX}_1{-}\textrm{RX}_1}$ and ${\textrm{TX}_{28}{-}\textrm{RX}_2}$ as *K* increases the KL is reduced. Also, for both the links ${K=1}$ yields the worst fit, where KL is 0.715 and 0.879, respectively. Furthermore, for ${\textrm{TX}_1{-}\textrm{RX}_1}$ the KL results of the MGs tend to stabilize for ${K\ge 15}$ and the best fit is achieved for ${K=17}$ with ${\textrm{KL}=0.019}$. Also, it is observed that the KL results for both the MGs and GMs for the ${\textrm{TX}_1{-}\textrm{RX}_1}$ link are similar for ${K\ge 3}$. For the ${\textrm{TX}_{28}{-}\textrm{RX}_2}$ link the MG KL results stabilize for ${K\ge 10}$ and the best fit is accomplished for ${K=20}$ with ${\textrm{KL}=0.087}$. The KL values of the GMs in Table 1 and those of the MGs in Fig. 2a denote that for both links, the MG yields a better fit than the GM. However, as shown in Fig. 2e, both the examined mixture distributions achieve an accurate fit to the empirical channel gain measurements. Meanwhile, in Fig. 2b, the RMSE for different values of *K* of the GMs and MGs for both the ${\textrm{TX}_1{-}\textrm{RX}_1}$ and ${\textrm{TX}_{28}{-}\textrm{RX}_2}$ links is depicted. According to Table 1, for both of the aforementioned links the maximum RMSE value is achieved for $K=1$. Meanwhile, for both the ${\textrm{TX}_1{-}\textrm{RX}_1}$ and ${\textrm{TX}_{28}{-}\textrm{RX}_2}$, the GM with $K=4$ yields the minimum RMSE, which is reported in Table 1. Also, Fig. 2b shows that for both ${\textrm{TX}_1{-}\textrm{RX}_1}$ and ${\textrm{TX}_{28}{-}\textrm{RX}_2}$ the RMSE values of the MGs are lower compared to those of the GMs. In more detail, the MG with ${K=17}$ yields the best fit to the empirical distribution of ${\textrm{TX}_1{-}\textrm{RX}_1}$ with ${\widehat{\textrm{R}}=-18.12\text { dB}}$. For the ${\textrm{TX}_{28}{-}\textrm{RX}_2}$ the best GM fit is accomplished for ${K=20}$ with ${\widehat{R}=-15.17\text { dB}}$.

Figure 2c and d serve as an illustrative example of the fitting achieved by the analytical GM expressions with different *K* to the empirical channel gain PDFs and CDFs for the links ${\textrm{TX}_1{-}\textrm{RX}_1}$ and ${\textrm{TX}_{28}{-}\textrm{RX}_2}$, respectively. Specifically, the blue circles represent the empirical channel gain distributions of the investigated links, while the continuous and dashed lines stand for the fitted GMs of different *K* for the links ${\textrm{TX}_1{-}\textrm{RX}_1}$ and ${\textrm{TX}_{28}{-}\textrm{RX}_2}$, respectively. Note that, unless otherwise is stated, the continuous and dashed lines of the same color denote GMs with the same *K*. By taking into account the KL and RMSE values of Table 1 and by examining the fitting of the PDFs and CDFs of the GMs to the empirical channel gain distributions of Fig. 2c and d, it can be ascertained that the increase of *K* leads to analytical GM expressions that better fit the empirical ones. Fig. 2e and f illustrate the fitting achieved by the analytical PDFs and CDFs of the GMs and MGs with different *K* to the empirical channel gain measurements of ${\textrm{TX}_1{-}\textrm{RX}_1}$ and ${\textrm{TX}_{28}{-}\textrm{RX}_2}$ links. In these figures, the blue circles represent the empirical channel gain PDFs and CDFs of the links. The continuous and dashed red and green lines stand for the GMs with *K* equal to 4 and 11, which denote the best fitting GMs to the empirical distributions according to the RMSE and KL metrics of the ${\textrm{TX}_1{-}\textrm{RX}_1}$ and ${\textrm{TX}_{28}{-}\textrm{RX}_2}$ links, respectively. Moreover, the red crosses indicate the MGs that yield the best fit to the empirical distributions according to both the metrics. In more detail, the MG with *K* equal to ${K_{\Gamma }=17}$ is the one that yields the best fit to the empirical distribution of ${\textrm{TX}_1{-}\textrm{RX}_1}$, whereas the MG with *K* equal to ${K_{\Gamma }=20}$ is the one that yields the best fit to the empirical distribution of ${\textrm{TX}_{28}{-}\textrm{RX}_2}$.

Figure 3 depicts the statistical characterization of the ${\textrm{TX}_4{-}\textrm{RX}_1}$ and ${\textrm{TX}_{16}{-}\textrm{RX}_1}$ links. In Fig. 3a, the KL values of GMs and MGs with different *K* for both ${\textrm{TX}_4{-}\textrm{RX}_1}$ and ${\textrm{TX}_{16}{-}\textrm{RX}_1}$ are presented. For the case of GM modeling, it is observed that, for both ${\textrm{TX}_4{-}\textrm{RX}_1}$ and ${\textrm{TX}_{16}{-}\textrm{RX}_1}$ as *K* increases KL is reduced. Up to ${K=7}$, KL presents a significant variation for both links. However, for ${K \in [8,20]}$, the resulting KL values stabilize. From Table 1, the minimum KL value for both the ${\textrm{TX}_4{-}\textrm{RX}_1}$ and ${\textrm{TX}_{16}{-}\textrm{RX}_1}$ links corresponds to a GM with ${K=15}$, whereas ${K=1}$ leads to the worst fit. For the case of MG modeling, it is observed that, for both ${\textrm{TX}_4{-}\textrm{RX}_1}$ and ${\textrm{TX}_{16}{-}\textrm{RX}_1}$ as *K* increases KL is reduced. For ${\textrm{TX}_4{-}\textrm{RX}_1}$, up to ${K=5}$, KL shows a significant variation, whereas for ${K\in [6,20]}$, the KL values stabilize. Also, according to the KL metric the MG that performs the best fit to ${\textrm{TX}_4{-}\textrm{RX}_1}$ is the one with ${K=20}$ and ${\textrm{KL}=0.003}$, whereas the worst fit is for the MG with ${K=1}$ and ${\textrm{KL}=2.152}$. By taking this into account, and according to the KL results for the GMs of Table 1, the MG yields a better fit to the empirical channel gain distribution of this link. For ${\textrm{TX}_{16}{-}\textrm{RX}_1}$, up to ${K=7}$ the KL results of MGs vary significantly, whereas, for ${K\in [8,20]}$, the KL values of the MGs tend to stabilize. Also, according to the KL metric the MG that yields the best fit for this link is the one with ${K=20}$ and ${\textrm{KL}=0.009}$, whereas the worst fit is obtained for ${K=1}$ and ${\textrm{KL}=1.856}$. From the KL values for the GMs of Table 1, it is observed that the MG achieves a better fit to the empirical channel gain measurements of ${\textrm{TX}_{16}{-}\textrm{RX}_1}$ in comparison with the GM. However, from Fig. 3a it is deducted that, both the GMs and MGs for the ${\textrm{TX}_4{-}\textrm{RX}_1}$ and ${\textrm{TX}_{16}{-}\textrm{RX}_1}$ link yield similar KL values for ${K\in [8,20]}$. In more detail, the good fit achieved by both the GMs and MGs to the empirical distributions of the investigated links is demonstrated by means of Fig. 3e and f. Meanwhile, Fig. 3b shows the RMSE metric results of the GMs and MGs for different values of *K* for both of the ${\textrm{TX}_4{-}\textrm{RX}_1}$ and ${\textrm{TX}_{16}{-}\textrm{RX}_1}$ links. For both ${\textrm{TX}_4{-}\textrm{RX}_1}$ and ${\textrm{TX}_{16}{-}\textrm{RX}_1}$ it is observed that as *K* increases the RMSE of the GMs is improved. However, for both the links the RMSE values for ${K\le 10}$ showcase significant variation. Table 1 reveals that, for both of the links $K=1$, yields the worst fitting accuracy performance, in terms of RMSE. Meanwhile, for ${\textrm{TX}_4{-}\textrm{RX}_1}$ the GMs with $K=15$, ${K=17}$, and ${K=20}$ yield $\mathrm {\widehat{R}}$ equal to $-19.28$, $-19.33$, and ${-19.37\text { dB}}$ respectively. For ${\textrm{TX}_{16}{-}\textrm{RX}_1}$ the resulting RMSE values almost stabilize for ${K\ge 15}$. For example, ${K=15}$, ${K=19}$, and ${K=20}$ yield ${\mathrm {\widehat{R}}=-18.07}$, ${\mathrm {\widehat{R}}=-18.12}$, and ${\mathrm {\widehat{R}}=-18.11\text { dB}}$, respectively.

From Fig. 3b, it observed that for the ${\textrm{TX}_4{-}\textrm{RX}_1}$ link the RMSE results of the MGs vary significantly for ${K\le 8}$ and improve with the increase of *K*. Meanwhile, based on the RMSE metric the MG with ${K=1}$ yields the worst fit, whereas the best fit is achieved for ${K=19}$ with ${\widehat{\textrm{R}}=-22.87}$. Furthermore, the RMSE metric results shown in Table 1 for the GMs and Fig. 3b demonstrate the better fitting accuracy of MGs compared to GMs for the empirical channel gain distribution of ${\textrm{TX}_4{-}\textrm{RX}_1}$. For the ${\textrm{TX}_{16}{-}\textrm{RX}_1}$ link as Fig. 3b illustrates the RMSE values of the MGs tend to stabilize for ${K\ge 11}$. The best fit for the link according to the RMSE is achieved for ${K=20}$ with ${\widehat{\textrm{R}}=-20.33\text { dB}}$, whereas the worst for ${K=1}$ with ${\widehat{\textrm{R}}=-8.57\text { dB}}$. Also, according to the RMSE values of the GMs for the ${\textrm{TX}_{16}{-}\textrm{RX}_1}$ link of Table 1 and Fig. 3b, the MGs yield a better fit to the empirical channel gain measurements of this link. Fig. 3c and d present the fitting accomplished by the analytical PDFs and CDFs of GMs with different *K* to the empirical channel gain distributions of the links ${\textrm{TX}_4{-}\textrm{RX}_1}$ and ${\textrm{TX}_{16}{-}\textrm{RX}_1}$. The blue circles represent the empirical channel gain distributions of the investigated links, while the continuous and dashed lines stand for the fitted analytical GMs of for ${\textrm{TX}_4{-}\textrm{RX}_1}$ and ${\textrm{TX}_{16}{-}\textrm{RX}_1}$, respectively. By taking into account the KL and RMSE values of Table 1 and by observing Fig. 2c and d, it can be ascertained that the increase of *K* leads to analytical GM expressions with improved fit to the empirical PDF and CDF. Moreover, it is obvious that a single Gaussian distribution (i.e. $K=1$) can not accurately describe the empirical data. Figures 3e and f illustrate the fitting achieved by the analytical PDFs and CDFs of the GMs and MGs with different *K* to the empirical channel gain distributions of the ${\textrm{TX}_4{-}\textrm{RX}_1}$ and ${\textrm{TX}_{16}{-}\textrm{RX}_1}$ links. In these figures, the blue circles stand for the empirical channel gain PDFs and CDFs of the ${\textrm{TX}_4{-}\textrm{RX}_1}$ and ${\textrm{TX}_{16}{-}\textrm{RX}_1}$. The continuous red and green lines denote the best fit achieved by the analytical GM to the empirical data of ${\textrm{TX}_4{-}\textrm{RX}_1}$ according to the RMSE and KL metrics, respectively, while the corresponding dashed lines denote the best fitting GM curves to ${\textrm{TX}_{16}{-}\textrm{RX}_1}$. Meanwhile, the curves marked with the red crosses and cyan dots indicate the analytical MGs that yield the best according to the RMSE and KL metrics to the empirical distribution of ${\textrm{TX}_4{-}\textrm{RX}_1}$ link with *K* equal to ${K_{\Gamma }=19}$ and ${K_{\Gamma }=20}$, respectively, while the red crosses with ${K_{\Gamma }=20}$ denote the MG that yields the best fit to ${\textrm{TX}_{16}{-}\textrm{RX}_1}$ according to both metrics. Figures 3e and f illustrate that both the GMs and MGs can yield a good fit to the data and can be both considered for the THz small-scale fading channel modeling.

Figure 4 presents the statistical characterization of ${\textrm{TX}_{25}{-}\textrm{RX}_2}$ link. In more detail, Fig. 4a shows the KL achieved by GMs and MGs with different *K*. For the GM it is observed that as *K* increases the KL improves. The value of $K=5$ yields ${\textrm{KL}=0.151}$, which is the first local minimum. Meanwhile, for ${K\ge 9}$ the KL stabilizes to almost the optimum value. For example, GMs with ${K=9}$, 14, 18, and 20 result to ${\textrm{KL}=0.131}$, 0.117, 0.106, and 0.112, respectively. Meanwhile, according to Table 1, $K=2$ yields the maximum value of KL and hence the worst fit. Moreover, from Fig. 4a it is observed that the MGs have similar performance with the GMs in terms of fitting when the KL metric is employed. The best fit of the MG is achieved for ${K=20}$, where ${\textrm{KL}=0.016}$. The similar fitting performance of GM and MG can also be observed in Fig. 4c and d. In Fig. 4b the RMSE for GMs and MGs with different *K* is presented. In more detail, for the GMs the first local minimum is obtained for ${K=4}$ and is ${\mathrm {\widehat{R}}=-11.18\text { dB}}$, while the second local minimum results for ${K=5}$ and is ${\mathrm {\widehat{R}}-13.1\text { dB}}$. Moreover, for ${K\ge 10}$ the RMSE almost stabilizes to the optimum value. For example, the GMs with $K=10$, 12, and 20 yield ${\mathrm {\widehat{R}}=-13.47}$, $-13.48$, and ${-13.4\text { dB}}$, respectively. Similar observations for the RMSE results of the MGs can be extracted as those for the GMs. However, according to this metric the MGs perform significantly better in terms of fitting for ${K\ge 13}$. The best fit is accomplished for the MG with ${K=20}$, where $\mathrm {\widehat{R}=-16.28\text { dB}}$.

In Fig. 4c and d the fitting achieved by the analytical PDF and CDF GM and MG expressions with different values of *K* to the empirical channel gain distribution of ${\textrm{TX}_{25}{-}\textrm{RX}_2}$ are presented. In more detail, the blue circles stand for the empirical distribution of the investigated link, whereas the continuous red, green and magenta lines indicate the GM with *K* equal to 4, 12 and 20, respectively. Also, the dashed black lines denote the analytical MG expressions obtained for *K* equal to ${K_{\Gamma }=20}$, which denotes the best fitting MG based on both metrics. Figure 4c and d illustrate that the best fit to the empirical data is accomplished by the GM with ${K=18}$, which is in accordance with the KL metric results. Also, it can be conducted that, in the case an empirical PDF with multiple peaks the increase of *K*, leads to a GM with a higher fitting accuracy performance. In this sense, the GM with $K=4$ performs the worst fit. As an example, for ${K=4}$ the metrics are ${\textrm{KL}=0.233}$ and ${\mathrm {\widehat{R}}=-11.18\text { dB}}$.

Figure 5 presents the statistical characterization of ${\textrm{TX}_{17}{-}\textrm{RX}_1}$ link. In more detail, Fig. 5a shows the KL achieved by GMs and MGs with different *K*. It is observed that, for the GMs as *K* increases the KL improves. In more detail, the GM with $K=5$ yields ${\textrm{KL}=0.139}$, which is the local minimum of the KL metric. Meanwhile, for ${K\ge 11}$ the KL results are almost equal. For example, for ${K=11}$, 15 and 20 the resulting KL is equal to 0.029, 0.03, and 0.026, respectively. Furthermore, based on Table 1, the GM with ${K=2}$ performs the worst fit in terms of the KL metric. Meanwhile, from Fig. 5a it is similarly observed that the increase of *K* improves the fitting accuracy of the MGs to the empirical channel gain data. Also, the KL results tend to stabilize for ${K\ge 10}$ and the best fit for the MG is accomplished for ${K=11}$, where ${\textrm{KL}=0.181}$. It should be noted that, according to the GMs KL metric of Table 1 the GM performs a better fit to the empirical data when compared to MG in terms of the KL metric. This significant difference is illustrated in Fig. 5c. In Figure 5b the RMSE for GMs and MGs with different *K* is presented. It is observed that, for the GMs the first and second RMSE local minima are ${\mathrm {\widehat{R}}=-14.77}$ and ${-17.89\text { dB}}$, which are obtained for a GM with ${K=5}$ and 9, respectively. The minimum RMSE according to Table 1 is accomplished for the GM with ${K=20}$. Meanwhile, as Fig. 5b illustrates as *K* increases the RMSE of the MGs improves and then deteriorates. This indicates that for the ${\textrm{TX}_{17}{-}\mathrm {RX_1}}$ link increasing the number of Gamma mixtures does not improve the fitting performance. The best fit in terms of the RMSE metric for the MG is achieved for ${K=11}$, where ${\mathrm {\widehat{R}}=-12.85\text { dB}}$. Both the KL and RMSE metrics shown in Fig. 5a and b denote that for an empirical distribution with multiple peaks the GM can yield a better fit in comparison with the MG.

Figure 5c and d illustrate the fitting achieved by the analytical PDF and CDF GMs and MGs of different *K* to the empirical channel gain measurements of ${\textrm{TX}_{17}{-}\textrm{RX}_1}$. In these figures, the blue circles stand for the empirical distributions, whereas the continuous red, green and magenta lines indicate the analytical PDFs and CDFs of the GMs with ${K=4}$, 12 and 20, respectively. Moreover, the dashed black lines stand for the MG obtained with *K* equal to ${K_{\Gamma }=11}$, which denotes the best fitting MG based on both metrics. Figure 5c and d demonstrate that the GM with ${K=20}$ yields the best fit. This can be verified by the KL and RMSE metric results of Table 1. Furthermore, it can be concluded that, in order to analytically describe an empirical distribution presenting multiple peaks a GM with a greater *K* is needed. As Figure 5c demonstrates the GM with ${K=4}$ achieves the worst fit to the empirical data.

**Table 1:** Fitting accuracy metrics of GMs with different values of *K*.

Link	d (m)	KL	${\widehat{\textrm{R}}\text { (dB)}}$	*K*	Link	d (m)	KL	${\widehat{\textrm{R}}\text { (dB)}}$	*K*
${\text{Tx}}_{1} - {\text{Rx}}_{1}$	13.72	0.191	$-12.61$	1	${\textrm{Tx}_{24} -\textrm{Rx}_2}$	57.11	0.267	$-10.93$	1
${\textrm{Tx}_{1} -\textrm{Rx}_1}$	–	0.037	${-\bf15.41}$	4	${\textrm{Tx}_{24} -\textrm{Rx}_2}$	–	0.038	${-\bf13.99}$	17
${\textrm{Tx}_{1} -\textrm{Rx}_1}$	–	**0.036**	$-15.35$	11	${\textrm{Tx}_{24} -\textrm{Rx}_2}$	–	**0.037**	$-13.98$	18
${\textrm{Tx}_{13} -\textrm{Rx}_1}$	16.41	0.097	$-14.7$	1	${\textrm{Tx}_{15} -\textrm{Rx}_1}$	58.68	0.597	$-11.44$	2
${\textrm{Tx}_{13} -\textrm{Rx}_1}$	–	**0.044**	$-16.64$	8	${\textrm{Tx}_{15} -\textrm{Rx}_1}$	–	**0.025**	${-\bf18.17}$	17
${\textrm{Tx}_{13} -\textrm{Rx}_1}$	–	0.045	${-\bf16.71}$	11	${\textrm{Tx}_{25} -\textrm{Rx}_2}$	57.71	0.932	$-7.25$	2
${\textrm{Tx}_{21} -\textrm{Rx}_2}$	19.71	0.238	$-11.68$	1	${\textrm{Tx}_{25} -\textrm{Rx}_2}$	–	**0.106**	$-13.4$	18
${\textrm{Tx}_{21} -\textrm{Rx}_2}$	–	**0.154**	${-\bf12.4}$	4	${\textrm{Tx}_{25} -\textrm{Rx}_2}$	–	0.126	$-\bf13.48$	12
${\textrm{Tx}_{8} -\textrm{Rx}_1}$	23.1	0.109	$-13.89$	2	${\textrm{Tx}_{18} -\textrm{Rx}_2}$	59.21	0.102	$-13.69$	1
${\textrm{Tx}_{8} -\textrm{Rx}_1}$	–	**0.058**	${-\bf15.38}$	20	${\textrm{Tx}_{18} -\textrm{Rx}_2}$	–	**0.058**	$-15.15$	6
${\textrm{Tx}_{2} -\textrm{Rx}_1}$	27.73	0.144	$-14.44$	1	${\textrm{Tx}_{18} -\textrm{Rx}_2}$	–	0.058	${-\bf15.22}$	16
${\textrm{Tx}_{2} -\textrm{Rx}_1}$	–	**0.026**	$-17.47$	15	${\textrm{Tx}_{4} -\textrm{Rx}_1}$	64.46	0.317	$-13.72$	1
${\textrm{Tx}_{2} -\textrm{Rx}_1}$	–	0.026	${-\bf17.5}$	19	${\textrm{Tx}_{4} -\textrm{Rx}_1}$	–	**0.019**	$-19.28$	15
${\textrm{Tx}_{20} -\textrm{Rx}_2}$	34.1	0.152	$-14.11$	1	${\textrm{Tx}_{4} -\textrm{Rx}_1}$	–	0.019	${-\bf19.37}$	20
${\textrm{Tx}_{20} -\textrm{Rx}_2}$	–	0.063	${-\bf15.59}$	7	${\textrm{Tx}_{32} -\textrm{Rx}_2}$	67.23	0.42	$-10.51$	1
${\textrm{Tx}_{20} -\textrm{Rx}_2}$	–	**0.062**	$-15.59$	9	${\textrm{Tx}_{32} -\textrm{Rx}_2}$	–	0.05	${-\bf14.12}$	13
${\textrm{Tx}_{30} -\textrm{Rx}_2}$	37.25	0.219	$-13$	1	${\textrm{Tx}_{32} -\textrm{Rx}_2}$	–	**0.046**	$-14.08$	20
${\textrm{Tx}_{30} -\textrm{Rx}_2}$	–	0.159	${-\bf13.6}$	8	${\textrm{Tx}_{11} -\textrm{Rx}_1}$	73.6	0.145	$-14.29$	1
${\textrm{Tx}_{30} -\textrm{Rx}_2}$	–	**0.158**	$-13.57$	12	${\textrm{Tx}_{11} -\textrm{Rx}_1}$	–	**0.025**	${-\bf18.42}$	4
${\textrm{Tx}_{14} -\textrm{Rx}_1}$	38.15	0.061	$-15.85$	1	${\textrm{Tx}_{5} -\textrm{Rx}_1}$	78.47	0.195	$-14.97$	1
${\textrm{Tx}_{14} -\textrm{Rx}_1}$	–	**0.018**	$-18.03$	7	${\textrm{Tx}_{5} -\textrm{Rx}_1}$	–	**0.035**	$-17.85$	14
${\textrm{Tx}_{14} -\textrm{Rx}_1}$	–	0.018	${-\bf18.09}$	10	${\textrm{Tx}_{5} -\textrm{Rx}_1}$	–	0.035	${-\bf17.88}$	20
${\textrm{Tx}_{27} -\textrm{Rx}_2}$	38.6	0.263	$-13.68$	1	${\textrm{Tx}_{16} -\textrm{Rx}_1}$	81.02	0.797	$-10.69$	1
${\textrm{Tx}_{27} -\textrm{Rx}_2}$	–	**0.064**	${-\bf15.61}$	17	${\textrm{Tx}_{16} -\textrm{Rx}_1}$	–	**0.015**	$-18.07$	15
${\textrm{Tx}_{9} -\textrm{Rx}_1}$	40.02	0.331	$-12.89$	1	${\textrm{Tx}_{16} -\textrm{Rx}_1}$	–	0.015	${-\bf18.12}$	19
${\textrm{Tx}_{9} -\textrm{Rx}_1}$	–	**0.063**	$-16.1$	5	${\textrm{Tx}_{23} -\textrm{Rx}_2}$	81.09	1.593	$-6.74$	1
${\textrm{Tx}_{9} -\textrm{Rx}_1}$	–	0.063	${-\bf16.11}$	11	${\textrm{Tx}_{23} -\textrm{Rx}_2}$	–	**0.156**	${-\bf10.83}$	20
${\textrm{Tx}_{19} -\textrm{Rx}_2}$	42.35	0.454	$-12.53$	2	${\textrm{Tx}_{17} -\textrm{Rx}_1}$	94.66	**0.026**	$-\bf18.85$	20
${\textrm{Tx}_{19} -\textrm{Rx}_2}$	–	**0.024**	$-\bf18.61$	20	${\textrm{Tx}_{12} -\textrm{Rx}_1}$	99.61	0.283	$-13.16$	1
${\textrm{Tx}_3 -\textrm{Rx}_1}$	45.19	0.39	$-13.69$	2	${\textrm{Tx}_{12} -\textrm{Rx}_1}$	–	0.065	${-\bf15.38}$	18
${\textrm{Tx}_3 -\textrm{Rx}_1}$	–	**0.024**	$-18.57$	18	${\textrm{Tx}_{12} -\textrm{Rx}_1}$	–	**0.064**	$-15.37$	20
${\textrm{Tx}_3 -\textrm{Rx}_1}$	–	0.024	$-\bf18.62$	19	${\textrm{Tx}_{22} -\textrm{Rx}_2}$	110.1	0.528	$-9.54$	1
${\textrm{Tx}_{31} -\textrm{Rx}_2}$	53.21	0.116	$-13.44$	1	${\textrm{Tx}_{22} -\textrm{Rx}_2}$	–	0.138	${-\bf12}$	12
${\textrm{Tx}_{31} -\textrm{Rx}_2}$	–	0.048	${-\bf14.78}$	8	${\textrm{Tx}_{22} -\textrm{Rx}_2}$	–	**0.135**	$-12$	19
${\textrm{Tx}_{31} -\textrm{Rx}_2}$	–	**0.046**	$-14.74$	9	${\textrm{Tx}_{18} -\textrm{Rx}_1}$	127.86	0.391	$-12.17$	1
${\textrm{Tx}_{10} -\textrm{Rx}_1}$	57.11	0.187	$-14.35$	1	${\textrm{Tx}_{18} -\textrm{Rx}_1}$	127.86	**0.064**	$-15.03$	10
${\textrm{Tx}_{10} -\textrm{Rx}_1}$	–	**0.015**	$-18.94$	12	${\textrm{Tx}_{18} -\textrm{Rx}_1}$	127.86	0.064	${-\bf15.05}$	20
${\textrm{Tx}_{10} -\textrm{Rx}_1}$	–	0.015	${-\bf18.98}$	13	-	–	–	-	–
${\textrm{Tx}_{28} -\textrm{Rx}_2}$	19.86	0.123	$-\bf13.82$	4	${\textrm{Tx}_{28} -\textrm{Rx}_2}$	19.86	**0.122**	$-13.78$	9

Significant values are in bold

## Discussion

The majority of the THz small-scale fading channel modeling works employ analytical distributions, such as Nakagami–m, Rayleigh, Rice, ${\alpha {-}\mu }$, and Weibull^7,27–29^. However, these distributions are capable of only describing single-peak fading channels. In this work, the suitability of modeling single and multiple peaks PDFs of outdoor THz channels in terms of GMs is investigated. Also, MGs are fitted to the empirical channel gain measurements of some indicative links. It is observed that, for both the cases of single and multiple peaks, empirical channel gain distributions the increase of *K* yields GMs and MGs that better fit the data. Accordingly, this is verified by the results of the KL and RMSE fitting accuracy metrics. In more detail, for all of the investigated links, for the lower values of *K*, the KL and RMSE fitting accuracy performance deteriorates. For most of the links, low values of *K* tend to yield significant variations to the KL and RMSE. On the other hand, for all of the examined links, as *K* increases beyond a specific value, the KL and RMSE fitting accuracy results tend to stabilize. This elucidates that, for any given link, the best fit is accomplished by a GM or MG with a particular value of *K* or higher. Hence, further increasing *K* is expected to make only a slight difference on the fitting performance of the GMs to the empirical distributions. Moreover, from the analytical GM and MG distributions illustrated in Figs. 2, 3, 4 and 5 and according to equations (4) and (7) the defining parameter for an analytical GM or MG to present significant peaks is the weight parameter *w* of its Gaussian or Gamma distribution coefficients. In more detail, as an example, for analytical GM distributions such as those presented in Figs. 2${\left( \textrm{c}\right) }$ and 3${\left( \textrm{c}\right) }$ for each *K* the differences of the *w* parameters are not significant. On the other hand, for analytical GM distributions such as those that are shown in Figs. 4${\left( \textrm{c}\right) }$ and 5${\left( \textrm{c}\right) }$, especially by increasing *K* there are *w* values that are greater compared to the rest. As a result, the corresponding Gaussian distribution coefficient with such a *w*, is more prominent in defining the peak amplitudes of the total GM. To demonstrate this, Table 2 presents the *w* parameter values for the ${\textrm{Tx}_1{-}\textrm{Rx}_1}$ and ${\textrm{Tx}_{25}{-}\textrm{Rx}_2}$ links. Moreover, the fitting accuracy statistics for the MGs employed in this work, verified that the MGs can model the small-scale fading amplitude of THz links. By comparing the fitting accuracy of the MGs and GMs for some indicative TX–RX links, it is observed that they both achieve a good fit to the empirical channel gain measurements. This observation verifies the previous technical works, where both the GMs and MGs were found suitable for THz channel modeling^25^. Meanwhile, the MG yields a better fit than the GM for the majority of the investigated links. However, the fitting accuracy of the GM is superior than that of the MG for links with multiple peaks with severe changes of amplitude. In more detail, as Fig. 5c illustrates and based on the KL and RMSE fitting accuracy tests, the GM yields an accurate fit with ${K=20}$ to the empirical PDF of ${\textrm{TX}_{17}{-}\textrm{RX}_1}$. On the other hand, the MG fails to yield a good fit to the data for ${K\le 20}$, where according to both the metrics the best fit of the MG is accomplished for ${K=11}$. As a consequence, the resulting analytical PDF and CDF MG expressions do not fit at all the empirical ones of the ${\textrm{TX}_{17}{-}\textrm{RX}_1}$ link. Finally, as a future work we intend to use more outdoor THz wireless measurements and compare the fitting achieved by Gaussian and Gamma mixtures to the empirical channel distributions.

**Table 2:** Weight parameters, *w* for the GMs with *K* equal to 2, 4, 11, 12, 18, and 20 for the ${\textrm{Tx}_1 -\textrm{Rx}_1}$ and ${\textrm{Tx}_{25} -\textrm{Rx}_2}$ links.

${\textrm{Tx}_{25} -\textrm{Rx}_2}$	${K=2}$	${K=4}$	${K=11}$	${K=20}$	${K=4}$	${K=12}$	${K=18}$
$${\textrm{Tx}_1 -\textrm{Rx}_1}$$							
$w_1$	0.35	0.069	0.02	0.01	0.2	0.06	0.05
$w_2$	0.65	0.23	0.03	0.01	0.22	0.06	0.05
$w_3$	–	0.34	0.03	0.03	0.27	0.07	0.05
$w_4$	–	0.36	0.05	0.03	0.31	0.07	0.05
$w_5$	–	–	0.09	0.04	–	0.08	0.05
$w_6$	–	–	0.1	0.04	–	0.08	0.05
$w_7$	–	–	0.12	0.05	–	0.08	0.05
$w_8$	–	–	0.13	0.05	–	0.09	0.05
$w_9$	–	–	0.13	0.05	–	0.09	0.05
$w_{10}$	–	–	0.16	0.06	–	0.1	0.05
$w_{11}$	–	–	0.16	0.06	–	0.11	0.05
$w_{12}$	–	–	–	0.06	–	0.11	0.06
$w_{13}$	–	–	–	0.06	–	–	0.06
$w_{14}$	–	–	–	0.06	–	–	0.06
$w_{15}$	–	–	–	0.06	–	–	0.06
$w_{16}$	–	–	–	0.06	–	–	0.06
$w_{17}$	–	–	–	0.06	–	–	0.06
$w_{18}$	–	–	–	0.06	–	–	0.06
$w_{19}$	–	–	–	0.07	–	–	–
$w_{20}$	–	–	–	0.07	–	–	–

## Methods

### Preprocessing of the measurement data

The wireless communication channel is expressed in terms of the product of one deterministic and one stochastic coefficient. The deterministic part encapsulates the large-scale effects of the propagation, i.e., the pathloss. The large-scale fading phenomena are time-invariant and remain unchanged during the wireless signal propagation. On the other hand, the stochastic channel coefficient expresses the small-scale fading characteristics of the channel, which are time and frequency dependent. The study of the small-scale fading behavior of RF wireless signals of is great importance, because it can cause unpredicted deep fades to the received signal power. As a consequence, to perform small-scale fading characterization of the channel, the effect of pathloss should be eliminated. The channel sounding performed in the outdoor campus measurements provides power angular delay profiles (PADPs) for each of the Tx–RX links. For any given link, the PADPs are expressed

1 $$\begin{aligned} \textrm{PADP}\left( \phi ,t\right) = \sum _{i=1}^{I} G P_i \delta \left( \phi -\phi _i\right) \delta \left( t-t_i\right) , \end{aligned}$$

where $\phi _i$, $P_i$ and $t_i$ stand for the azimuth angle at the Rx, the propagation delay gain and time of the $i\text {--th}$ propagation path, respectively. The parameter *G*, known as the broadside angle, denotes the combined gains of the Tx and Rx antennas, while $\delta \left( \cdot \right)$ and *I* are the Dirac delta function and the total number of multipath components of a link, respectively. Subsequently, in order to eliminate the deterministic phenomenon of pathloss, by employing (1) to each link, the link pathgain measurements are normalized to unity as

2 $$\begin{aligned} \zeta _i^2=\frac{P_i}{\frac{\sum _{i=1}^{I} P_i}{I}}. \end{aligned}$$

### Incrementing a link channel realizations

The inherent high frequencies of the THz band lead to much higher propagation losses in comparison with the lower mmWave and ultra-high-frequency (UHF) bands^15,17,46^. The THz free space pathloss even at distances of a few meters and a low transmission frequency can be severe. As an example, for an operational frequency of ${140\text { GHz}}$ and a communication distance of ${1\text { m}}$ the free space pathloss can be in the excess of $80\text { dB}$^17,47^. Moreover, the atmospheric water vapor causes severe attenuation to the propagating THz signal^7,15^. Also, the wavelength of the emitted THz signal can be much smaller compared to the size of obstacles laid within the propagation environment^48^. As a consequence, the refraction and reflection losses of the THz band are significantly stronger, when compared to lower frequency bands^46,49–51^. This leads to a significant reduction of the number of dominant rays, since the THz signal power is drastically weakened, when it is reflected or scattered two or more times^48,49^. In this sense, the ability of the THz electromagnetic wave to propagate through blockages is nearly lost, due to the severe penetration loss. As a result, the ability of THz signals to diffract around obstacles is significantly reduced. The aforementioned remarks elucidate that, the THz band yields non-rich multipath environments, when compared for example to the mmWave band. However, still there are surfaces that can act as scatterers for propagating wireless THz signals^16,17,20,29,46^. This leads to the existence of reflected NLoS multipath components carrying a significant amount of power, which are capable of being detected by the Rx. Nevertheless, the amount of measured multipath components, utilized in our analysis, is still not adequately enough to perform small-scale fading statistics analysis for a THz wireless channel. This limitation is surpassed by generating different realizations of the transfer function. This is accomplished by changing the phases of the measured multipath components of a link^7,45^. The random phases are assumed to be stochastic and are given by a uniform distribution in the interval ${[0,2\pi ]}$. This assumption is based on the contribution of Molisch et. al, which was based on the principle that the aggregated phases of different paths in an environment of moving scatterers followed a uniform distribution^45^. Hence, from the electromagnetic theory point of view, this is extracted by taking into account the phase shift due to the Doppler effect and it stands in any propagation environment where motion is present. The channel coefficient of the system can be obtained as^7,45^

3 $$\begin{aligned} h=\sum _{i=1} \zeta _i \exp \left( -j2\pi f t_i\right) \exp (j \psi _i), \end{aligned}$$

where ${\psi _i\ \sim U\left( 0,2\text { }\pi \right) }$ represents the random phase of the $i\text {-th}$ multipath component. Moreover, by assuming that the amplitude of the channel coefficients does not change dramatically among the progressing $t_i$, i.e., the channel can be considered as flat-fading then, ${t_i=0}$^45^. Also, the term ${U\left( \cdot ,\cdot \right) }$ is the uniform distribution operator^52^.

### Expectation-maximization based fitting approach

#### The gaussian and gamma mixture models

The THz small-scale fading phenomenon has been the epicenter of many recent channel modeling studies^7,25,27,51^. Moreover, it has been experimentally observed that there are wireless THz propagation scenarios, where the small-scale fading channel amplitude shows significant fluctuations^25^. In this sense, the commonly used analytical distributions that are only capable of fitting single peak distributions are now inadequate to describe the small-scale fading amplitude of such THz channels. However, by considering small-scale fading THz and lower frequency studies, mixture distributions such as Gaussian and Gamma can be employed instead^25,35,38,53^.

The GMs have been extensively employed to describe the small-scale fading channel amplitude of RF wireless channels^25,35,38^. The PDF of the GM is defined as

4 $$\begin{aligned} f_{gm}\left( x\right) =\sum _{i=1}^{K}w_i \frac{\exp \left( -\frac{\left( x-\mu _i\right) ^2}{2\sigma _i^2}\right) }{\sqrt{2 \pi }\sigma _i}, \end{aligned}$$

where *K* and $w_i$ denote the number of GM components and the weight of the ${i\text {-th}}$ mixture component, respectively. The parameters $\mu _i$ and $\sigma _i$ stand for the mean and standard deviation of the $i\text {-th}$ GM component, respectively. Also, ${w_i\in [0,1]}$ and

5 $$\begin{aligned} \sum _{i=1}^{K}w_i=1. \end{aligned}$$

The CDF of the GM is expressed as

6 $$\begin{aligned} \textrm{F}_{gm}\left( x\right) =\frac{1}{2} \sum _{i=1}^{K} w_i Erfc\left( \frac{\mu _i -x}{\sqrt{2} \sigma _i }\right) , \end{aligned}$$

where ${Erfc\left( \cdot \right) }$ is the complementary error function^41^. Moreover, of note is the fact that the *K* Gaussian distributions that comprise equation (4) are mutually independent. Hence, the GM is not only a favorable distribution for modeling significant empirical distribution amplitude fluctuations, but also it can offer analytical tractability. The latter is of great importance, when the performance analysis of a wireless system must evaluated. Also, it should be noted that since this work employs pathloss measurements the *x* instance of a GM is always non-negative, hence for the PDF of equation (4) ${x\in [0,\infty )}$.

The MGs have been employed in various channel modeling works in lower frequency bands and the THz band as well^25,54,55^. The PDF of the MG is defined as

7 $$\begin{aligned} f_{\Gamma m}\left( x\right) =\sum _{i=1}^{K} w_i \frac{1}{{b_i}^{a_i} \Gamma \left( a_i\right) } x^{a_i-1} \exp \left( -\frac{x}{b_i}\right) , \end{aligned}$$

where $a_i$ and $b_i$ stand for the shape and scale parameters of the $i\text {-th}$ MG component. Also, according to the definition of equation (7) ${x\in [0,\infty )}$ and the operator $\Gamma \left( \cdot \right)$ denotes the gamma function^41^. The CDF of the MG is defined as

8 $$\begin{aligned} \textrm{F}_{\Gamma m}\left( x\right) = \sum _{i=1}^{K} w_i \frac{1}{\Gamma \left( a_i\right) } \gamma \left( a_i,\frac{x}{b_i}\right) , \end{aligned}$$

where ${\gamma \left( \cdot ,\cdot \right) }$ stands for the lower incomplete gamma function^41^.

### The expectation maximization algorithm

The weights and the parameters of the Gaussian distributions that compose the GM with the best possible fit to the empirical data must be identified by employing an appropriate method. The EM algorithm is such a method. The EM is a machine learning approach that simplifies maximum-likelihood-estimate (MLE) problems and is vastly used in calculating the parameters of mixture models^25,35^.

The EM is a two step algorithm. It consists of the expectation (E) and the maximization (M) steps^40^. To operate the EM algorithm, the *K* number of mixtures and the vector ${\textbf{y}=\left( y_1,...,y_n\right) }$ of the *n* channel gain measurements of a link are required as inputs. Subsequently, the mixtures parameters are updated at the M–step during the ${m+1}$ iteration of the EM algorithm until the convergence criterion is met. Otherwise the EM terminates, when a predefined number of repetitions is reached. The converge criterion is defined as

9 $$\begin{aligned} \left| \textrm{L}^{[m+1]}-\textrm{L}^{[m]}\right| >\varepsilon , \end{aligned}$$

where $\varepsilon$ stands for the desired convergence value. The term ${\textrm{L}^{[m]}}$ signifies the MLE log–likelihood at the ${m\text {-th}}$ iteration of the EM algorithm and can be obtained as

10 $$\begin{aligned} \textrm{L}^{[m]}=\frac{1}{n}\sum _{i=1}^{n}\textrm{ln}\left( \sum _{j=1}^{K}w_j^{[m]} \phi \left( y_i \Bigg | \mu _j^{[m],\sigma _j^{[m]}} \right) \right) , \end{aligned}$$

where ${j\in [1,K]}$, ${i\in [1,n]}$ and ${\textrm{ln}\left( \cdot \right) }$ stands for the natural logarithm. The term ${\phi \left( y_i \Bigg | \mu _j^{[m],\sigma _j^{[m]}} \right) }$ is the Gaussian distribution of the ${j\text {-th}}$ mixture component at the ${m\text {-th}}$ iteration of the EM, which has mean and standard deviation ${\mu _j^{[m]}}$ and $\sigma _j^{[m]}$, respectively. Meanwhile, the E–step of the EM is implemented as

11 $$\begin{aligned} \gamma _{ij}^{[m]}=\frac{w_j^{[m]} \phi \left( y_i \Bigg | \mu _j^{[m]},\sigma _j^{[m]}\right) }{\sum _{l=1}^{K}w_l^{[m]} \phi \left( y_i \Bigg | \mu _l^{[m]},\sigma _l^{[m]}\right) }. \end{aligned}$$

Uppon the completion of the E–step, the EM algorithm implements the M–step. The M–step provides the updated values of the distribution parameters of the ${j\text {-th}}$ mixture at the ${m+1}$ step of the algorithm, which for the particular case of a GM are calculated as in equations (12)–(14)

12 $$\begin{aligned} w_j^{[m+1]}=\frac{1}{n}\sum _{i=1}^{n} \gamma _{ij}^{[m]}, \end{aligned}$$

13 $$\begin{aligned} \mu _j^{[m+1]}=\frac{\sum _{i=1}^{n} \gamma _{ij}^{[m]}y_i}{\sum _{i=1}^{n}\gamma _{ij}^{[m]}}, \end{aligned}$$

14 $$\begin{aligned} \sigma _j^{[m+1]}=\sqrt{\frac{\sum _{i=1}^{n} \gamma _{ij}^{[m]}\left( y_i-\mu _j^{[m+1]}\right) ^2}{\sum _{i=1}^{n} \gamma _{ij}^{[m]}}}. \end{aligned}$$

The convergence of the EM algorithm depends on *K* and the initialization values of the mixtures parameters that are provided as inputs. Several methods are available to provide initialization values for the mixtures parameters. One of the most common is to employ the K-nearest-neighbour (KNN) algorithm^56^.

### Evaluation of the fitting

#### The kolmogorov-smirnov test

The KS goodness of fit test is defined as^41^

15 $$\max \left( {\left| {F_{{emp}} \left( x \right) - F_{{gm}} \left( x \right)} \right|} \right) \le \sqrt { - \frac{1}{{2N}}\ln \left( {\frac{A}{2}} \right)} ,$$

where $F_{emp}\left( x\right)$ and *N* stand for the empirical values of the channel gain CDF of the examined link and the number of discrete samples of $F_{emp}\left( x\right)$, respectively. The parameter $F_{gm}\left( x\right)$ denotes the analytical CDF of the examined analytical distribution, while $A=5\%$ is the selected significance level.

#### Kullback–leibler divergence test

The KL divergence test is defined as the distance between the empirical PDF $f_{emp}\left( x\right)$ and the analytical PDF $f_{gm}\left( x\right)$ of the examined distribution i.e.,^42^

16 $${\text{KL}} =  - \sum\limits_{{i = 1}}^{N} {f_{{emp}} } \left( {x_{i} } \right)\ln \left( {\frac{{f_{{gm}} \left( {x_{i} } \right)}}{{f_{{emp}} \left( {x_{i} } \right)}}} \right) $$

The closer the value of equation (16) to 0 the better is the fit of the analytical fading distribution to the empirical channel gain distribution.

#### The root mean square error

The RMSE is defined as^43^

17 $$\begin{aligned} \mathrm {\widehat{R}}=\sqrt{\frac{1}{N}\sum _{i=1}^{N}\left( f_{emp}\left( x_i\right) -f_{gm}\left( x_i\right) \right) ^2}. \end{aligned}$$

The lower the value of ${\mathrm {\widehat{R}}}$ the better the fit of the analytical $f_{gm}\left( x\right)$ PDF to the empirical distribution. Also, it should be noted that the RMSE results are commonly presented in dB scale.

## Supplementary Information

Supplementary Information.

## Data Availability

The initial data are owned by Aalto University Finland. Any researcher affiliated to one of the ARIADNE project partners is allowed to access and use the shared data for research purposes. The shared data must however not be made accessible to any person not affiliated with an ARIADNE project partner. All the processed data present in this work accompany this manuscript as a supplementary material.
